# Mackenzie and the German Doctors

**Published:** 1888-11

**Authors:** 


### MACKENZIE AND THE GERMAN DOCTORS.

As nearly all, if not every single medical journal in the world —so far as we know—have had something to say in the line of advertising "Sir" Dr. Mackenzie, we feel called upon to chip in our share, for consistency's sake, and to let people know that this JOURNAL *has* an opinion on the subject, as well as on every other subject, and that we are not afraid to express it.

"Sir" Dr. Mackenzie is certainly the best advertised doctor in the two continents, towards which end he contributed not a little himself. Well, we can hardly blame him for doing a little blowing, under the circumstances, since we condone the same thing on this side, and wink at an "interview" with an advertising phase for the doctor interviewed. But, when he details his conversation with the emperor, and prints the identical boot-licking, servile language addressed to his Majesty, such, for instance, as "I fear, your Majesty, that if Dr. Bergman is permitted to again touch your Majesty's majestic throat, Sir, I cannot have the honor of continuing to serve your Majesty longer, your Majesty," or words to that effect,—ugh, it makes us tired! It is only excelled by "Bill's" boast that he "seen Phil. Armour, and shuck han's with him!" But we exonorate the doctor from all censure—from everything, for having come out, like a man, and "told the truth;" told of the utter incompetency, not to say brutality, of those swell professors with "Herr" to the handle of their names. If what he says of Dr. Bergmann's awkward attempts and utter failure to introduce the canula be true, and that he had to send for Braman, his young assistant, to do it, there is a strong suspicion that Bergmann was drunk.

At first we felt for the German Doctors ; felt that the Emperor was almost a brute to send to the enemy's country for a doctor, when (we thought) the woods were full of better doctors in Germany. We had been led to this belief, partly by the persistent course pursued by certain would-be leading journals in America, in quoting, under the head of "Medical Progress" everything "German" and nothing from any thing American, as Brother Lanphear points out in his "Index,—or we believed Fred, the noble, was a weak brother, to let his English wife persuade him into so discourteous an act, discourteous to the German profession, as to send for her Ma's doctor from across the channel. Suppose, when Garfield was wounded, for instance, that he had repudiated the entire profession of America, (pity he did not have a little more penetration and discretion and kick out the bold blunderer Bliss,) and had sent for an English or German surgeon, and had given him full charge,—as Mackenzie seems to have had,—what would ye American have done or said? Would they not have been as jealous as Turks? Hence, we say, we felt for the Germans,—that they had been badly treated.

But, now we think we understand it,—thanks to "Sir" Dr. Mackenzie. The Emperor knew Bergmann and the whole compoodle. If Frederick was the great man everybody says he was, it is impossible for him not to have become aware during more or less intimate acquaintance with the surgeons, that they were *ignorant*, comparatively ; it is impossible for the shrewdest person, to conceal his ignorance in the presence of one whose perceptive faculties were as keen as were those of the illustrious Frederick ; *and Bergmann knew it*, hence his embarrassment. We owe Mackenzie one !

---

THE NORTH TEXAS MEDICAL ASSOCIATION will meet in Sherman, Texas, December 11, 12 aud 13, prox. Dr. J. T. Wilson, of Sherman, is president, and Dr. Geo. R. Clayton, is secreary. The programme for the meeting is as follows:

Section on Surgery: Recent Progress in Surgery, by F. D. Thompson, M. D., of Sherman. Hemorrhage, by A. C. Scott, M. D., of Gainesville. Pathology and Treatment of Surgical Fever, by W. R. Mathers, M. D., of Collin county.

Obstetrics and Gynecology: Troubles Connected with the Menopause, by I. P. Gumby, M. D., of Sherman. Significance of Uterine Hemorrhage, by J. C. Erwin, M. D., of McKinney. Dysmenorrhœa, by S. C. Ball, M. D., of New Boston, Bowie county.

Practice of Medicine: Acute Bright's Disease, by S. D. Moore, M. D., of Van Alstyne. Dysentery, by J. J. Dial, M. D., of Sulphur Springs. Diptheria, by J. B. Hill, M. D., of Collin county.

Volunteer papers will also be read.

